# The right hemispheric dominance for face perception in preschool children depends on the visual discrimination level

**DOI:** 10.1111/desc.12914

**Published:** 2019-11-15

**Authors:** Aliette Lochy, Christine Schiltz, Bruno Rossion

**Affiliations:** ^1^ Cognitive Science and Assessment Institute Education, Culture, Cognition, and Society Research Unit University of Luxemburg Esch‐sur Alzette Luxembourg; ^2^ IPSY Université Catholique de Louvain Louvain‐La‐Neuve Belgium; ^3^ CNRS CRAN Université de Lorraine Nancy France; ^4^ CHRU‐Nancy Université de Lorraine Nancy France

**Keywords:** discrimination level, faces, FPVS–EEG, preschool children, right hemisphere

## Abstract

The developmental origin of human adults’ right hemispheric dominance in response to face stimuli remains unclear, in particular because young infants’ right hemispheric advantage in face‐selective response is no longer present in preschool children, before written language acquisition. Here we used fast periodic visual stimulation (FPVS) with scalp electroencephalography (EEG) to test 52 preschool children (5.5 years old) at two different levels of face discrimination: discrimination of faces against objects, measuring face‐selectivity, or discrimination between individual faces. While the contrast between faces and nonface objects elicits strictly bilateral occipital responses in children, strengthening previous observations, discrimination of individual faces in the same children reveals a strong right hemispheric lateralization over the occipitotemporal cortex. Picture‐plane inversion of the face stimuli significantly decreases the individual discrimination response, although to a much smaller extent than in older children and adults tested with the same paradigm. However, there is only a nonsignificant trend for a decrease in right hemispheric lateralization with inversion. There is no relationship between the right hemispheric lateralization in individual face discrimination and preschool levels of readings abilities. The observed difference in the right hemispheric lateralization obtained in the same population of children with two different paradigms measuring neural responses to faces indicates that the level of visual discrimination is a key factor to consider when making inferences about the development of hemispheric lateralization of face perception in the human brain.

## Graphical abstract

Using a fast periodic visual presentation paradigm combined with EEG we show that the right hemisphere involvement in face processing depends on discrimination level in preschool children. While face individuation (identity) relies on the right hemisphere, bilateral occipital networks were activated by generic face categorization (faces vs objects). Since right lateralization is present in pre‐readers these results challenge the hypothesis that learning to reading entails the right‐lateralization for faces observed in adulthood.

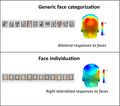

**Research highlights**

- Fast periodic visual presentation with electroencephalography (EEG) recordings reveals that the right hemisphere involvement in face processing depends on the discrimination level in 52 preschool children.
- Face individuation (identity) relies on the right hemisphere, generic face categorization (faces vs. objects) relies on bilateral occipital networks.
- This finding challenges the view that the right lateralization for faces causally depends on learning to read, as it is already present in pre‐readers.
- Preschoolers show a reduction of amplitude for individual discrimination of inverted faces, this effect being much smaller than in adults.

## 1 INTRODUCTION

Neurotypical human adults have an astonishing ability to recognize the identity of people from their faces, often at a single glance and automatically. A wide variety of evidence supports a right hemispheric dominance in this function. For instance, damage to the ventral occipitotemporal cortex bilaterally or in the right hemisphere only may lead to prosopagnosia – a rare inability to specifically recognize individual faces following brain damage (Meadows, 1974; Rossion, 2018 for recent review). Differential stimulation in the left and right visual field has also pointed to a right hemisphere advantage in individual face recognition (e.g., Hillger & Koenig, 1991). Neuroimaging (Kanwisher, McDermott, & Chun, 1997; Sergent, Ohta, & MacDonald, 1992) and high‐density electroencephalographic (EEG) recordings on the human scalp (e.g., Bentin, Allison, Puce, Perez, & McCarthy, 1996; Rossion, Torfs, Jacques, & Liu‐Shuang, 2015) have also reported higher amplitudes of neural responses to faces in the right than the left hemisphere. More recently, a strong right hemispheric dominance for face‐selective responses in the human ventral occipitotemporal cortex (VOTC) has been reported with intracerebral electrophysiological recordings (Jonas et al., 2016), with several regions in the right but not the left hemisphere being causally related to (individual) face perception defects (Jonas et al., 2012, 2015; Parvizi et al., 2012).

The developmental origin of the right hemispheric lateralization for face perception, which is specific to the human species (Rossion & Taubert, 2019), remains largely unknown and debated. On the one hand, the right hemispheric specialization for face perception may emerge relatively *early* during development, that is, being present already at a few months of age (de Schonen & Mathivet, 1989). This proposal is based on the observation that 4‐ to 9‐month‐old infants saccade faster toward their mother's face than a stranger's face when these pictures are presented in the left visual field (LVF) but not in the right visual field (RVF; de Schonen, Gil de Diaz, & Mathivet, 1986; de Schonen & Mathivet, 1990). Along the same line, the right hemisphere but not the left hemisphere early deprivation of visual input for several months (between 6 weeks and 3 years) impairs the development of the adult face processing system (Le Grand, Mondloch, Maurer, & Brent, 2003). At the neural level, while a number of studies using EEG or neuroimaging in infants have failed to find clear hemispheric differences in face perception in infancy (de Haan & Nelson, 1999; Gliga & Dehaene‐Lambertz, 2007; Tzourio‐Mazoyer et al., 2002), functional near‐infrared spectroscopy (fNIRS) studies have often shown a significant right hemisphere (RH) advantage for faces over control visual stimuli in 5‐ to 8‐month‐old infants (e.g., Otsuka, 2014; for review). More recently, a robust face‐selective frequency‐tagged EEG response not accounted for by low‐level visual cues has been observed predominantly over the right occipitotemporal cortex already at 4–6 months of age (de Heering & Rossion, 2015). The same approach tested in 9 months old reveal a right hemispheric dominance for discriminating pictures of human faces from monkey faces and even for individuating monkey faces (Barry‐Anwar, Hadley, Conte, Keil, & Scott, 2018; Peykarjou, Hoehl, Pauen, & Rossion, 2017, respectively). In addition, an EEG study with lateralized stimulus repetition showed individual discrimination of faces only in the RH in 1‐ to 5‐month‐old infants (Adibpour, Dubois, & Dehaene‐Lambertz, 2018). Altogether, these observations generally support the view that the right hemisphere takes precedence over the left hemisphere for face perception at an early age, perhaps due to a faster maturation rate of the right hemisphere at a time at which the infants’ visual system mainly extracts low spatial frequencies carrying global information from facial inputs (de Schonen & Mathivet, 1989; Sergent, 1982).

On the other hand, the right hemispheric lateralization (generally) observed in infants for face perception does not appear to be carried out uniformly throughout development until adulthood. Indeed, with a few exceptions (Cantlon, Pinel, Dehaene, & Pelphrey, 2011), EEG and fMRI studies generally report bilateral responses to faces in children. For instance, small face‐selective fMRI responses are bilateral in 5‐ to 8‐year‐old children (Golarai et al., 2007; Natu et al., 2016; Scherf, Behrmann, Humphreys, & Luna, 2007), they progressively enlarge in older children with small right lateralization effects (7–11 years old, Gathers, Bhatt, Corbly, Farley, & Joseph, 2004; Golarai et al., 2007; Natu et al., 2016; Peelen, Glaser, Vuilleumier, & Eliez, 2009) that increase slowly between childhood and adolescence (Cohen Kadosh, Cohen Kadosh, Dick, & Johnson, 2011; Joseph, Gathers, & Bhatt, 2011). Likewise, the N170 does not show significant lateralization in children of various age groups until late adolescence (Dundas, Plaut, & Behrmann, 2012, 2014; Kuefner, de Heering, Jacques, Palmero‐Soler, & Rossion, 2010). Importantly, although these differences across age could be attributed to the use of different paradigms and stimuli across studies, the very same EEG frequency‐tagging paradigm showing a strong right hemispheric lateralization of face‐selective responses in 4‐ to 6‐month‐old infants (de Heering & Rossion, 2015) as well as in adults (Rossion et al., 2015) elicits a completely bilateral response in preschool children (Lochy, de Heering, & Rossion, 2019). Such observations can be taken in support of the view that the right hemispheric specialization for face perception observed in adulthood emerges – or rather *re*emerges – relatively late in development. A proposed key factor for this relatively late right hemispheric dominance is the gradual specialization of the left ventral occipitotemporal cortex to written script during reading acquisition, this specialization competing with the representation of faces in the left hemisphere and therefore causally driving faces to be dominantly processed by the right hemisphere (Behrmann & Plaut, 2013; Dehaene, Cohen, Morais, & Kolinsky, 2015).

A potentially important factor that has been neglected regarding this issue, and in neurodevelopmental studies in general, concerns the visual categorization or discrimination *level* of the presented face stimuli. At a very coarse level, a face is categorized as a face by comparison to other stimuli, that is, nonfaces objects. At the finest level, a face has to be discriminated from other faces to give access to its identity. Developmental studies either measure the *absolute* neural response to faces (even when a same‐different matching task is used, Dundas et al., 2014) or a face‐selective response, that is, a difference between faces and nonface stimuli (Cantlon et al., 2011; Gathers et al., 2004; Golarai et al., 2007; Scherf et al., 2007; see Natu et al., 2016 for fMRI measures of individual face discrimination in children, but without separating left and right hemisphere responses). However, the right lateralization of the face processing function in adults generally concerns the *individuation of faces*. Brain‐damaged patients with prosopagnosia, for instance, cannot individuate familiar or unfamiliar faces but can still recognize a face as a face (e.g., Liu‐Shuang, Torfs, & Rossion, 2016; Rossion, Dricot, Goebel, & Busigny, 2011; Young, De Haan, & Newcombe, 1990). Likewise, transcranial magnetic stimulation over the right lateral occipital cortex impairs individuation of faces (Ambrus, Dotzer, Schweinberger, & Kovács, 2017; Pitcher, Walsh, Yovel, & Duchaine, 2007; Solomon‐Harris, Mullin, & Steeves, 2013) but not the categorization of a face as a face (Solomon‐Harris et al., 2013). In the same vein, transient failures to individuate faces have been observed following intracranial stimulation in the right but not the left face‐selective regions (Jonas et al., 2012, 2015), while difficulties at categorizing visual stimuli as faces can be observed following stimulation in either the left or right hemisphere face‐selective regions (Chong et al., 2013; Keller et al., 2017). In EEG studies, indexes of individual face discrimination usually found using stimulus repetition are strongly right lateralized, whether they are obtained in standard ERP designs (i.e., on the N170, Jacques & Rossion, 2007; Rossion, 2009) or with EEG frequency‐tagging (Liu‐Shuang, Norcia, & Rossion, 2014; Rossion & Boremanse, 2011). In contrast, differences between faces and objects are less strongly and less systematically right lateralized (Rossion et al., 2015).

On this basis, we reasoned here that the apparent lack of right hemispheric lateralization for face stimuli in young children may be due to the lack of a diagnostic neural measure of individual face discrimination and, more generally, that the pattern of hemispheric lateralization in children may depend on the level of visual discrimination tested. Therefore, our main objective was to test hemispheric lateralization of the response to faces in the same group of preschool children at two levels of discrimination (faces vs. objects, and individual faces vs. other individual faces).

**Figure 1:**
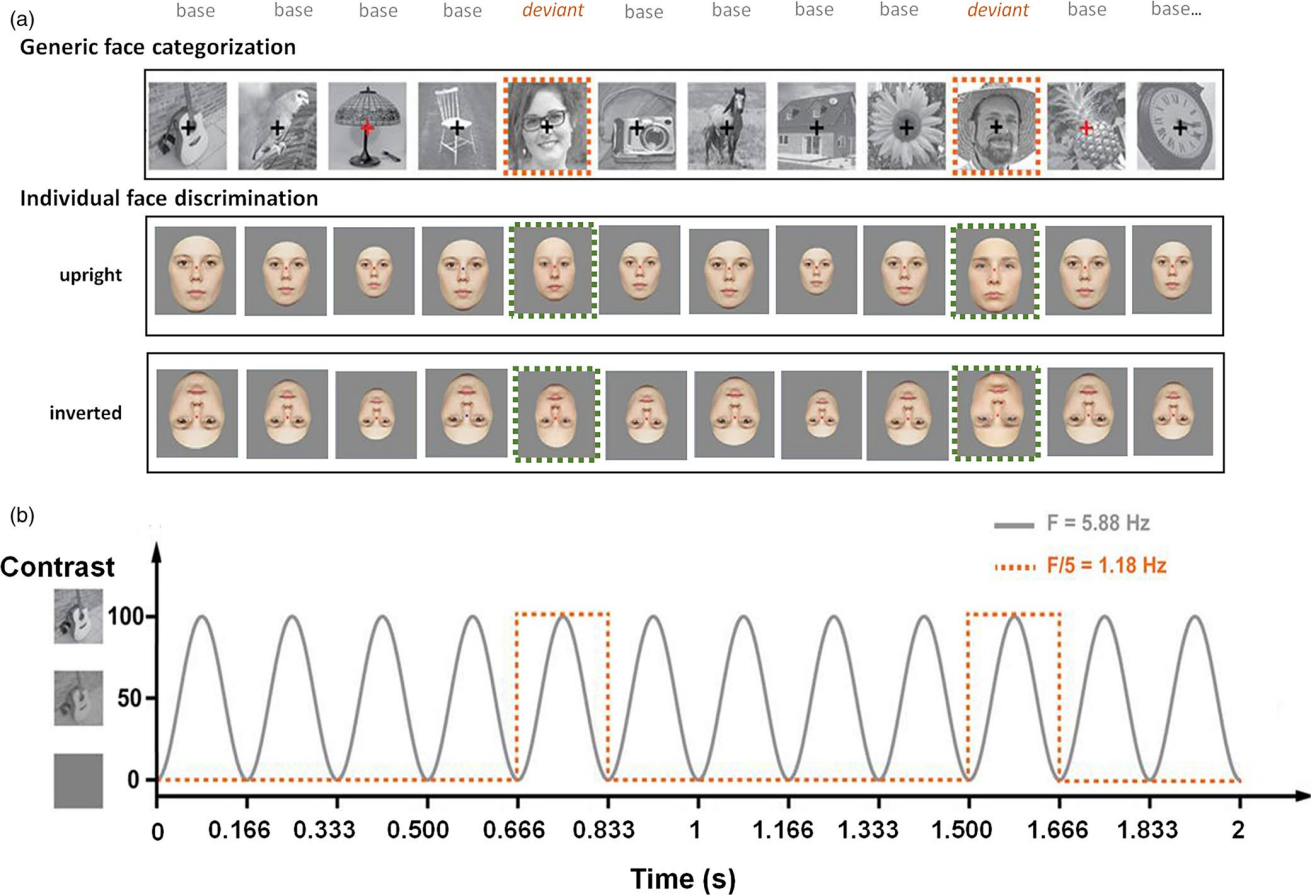
Experimental design. (a) Stimulation sequences used in the two paradigms. In the generic face categorization paradigm (first row), base stimuli are constituted of various nonface objects, and faces are inserted every five items (various identities, backgrounds, etc). In the individual face discrimination paradigm (last two rows), base stimuli are constituted of the same individual face, and every five items other identities are inserted. Faces are shown either in upright (middle row) or in inverted orientation (last row). (b) Stimulation mode: Six stimuli were presented per second with a sinusoidal contrast modulation, and every fifth item was the periodic deviant stimulus. Two sequences of 40 s were recorded by condition

To do so, we used EEG frequency‐tagging, or fast periodic visual stimulation (FPVS), in which visual stimuli appear at a fast rate (6 Hz, 6 images/s) during 40 s. In this stream of images, deviant images are inserted every five items, thus at 6 Hz/5, that is, 1.2 Hz. This presentation mode allows recording responses exactly at the frequency of the deviant stimuli, that is, at 1.2 Hz (and harmonics), if they are reliably (i.e., periodically) discriminated from the base visual stimuli (Liu‐Shuang et al., 2014; Rossion et al., 2015). In the generic face categorization paradigm, recently reported in a smaller sample of 5‐year‐old children (*N* = 35; Lochy et al., 2019), different faces are presented (with different backgrounds, orientations, ages, skin color, etc.) among streams of natural objects (houses, flowers, animals, etc.). Therefore, a discrimination response reflects not only discrimination of faces versus other objects but also generalization of this response across the different faces (Rossion et al., 2015). In the individual face discrimination paradigm, the base images are constituted of an unfamiliar face identity presented repeatedly, varying in size. The deviant faces are different unfamiliar identities (Liu‐Shuang et al., 2014; see Liu‐Shuang et al., 2016; Vettori et al., 2019 for direct comparison of the two paradigms). In this latter paradigm, external features are removed from the pictures (hair, ears, etc.), faces all have a neutral expression and the same gender, therefore constraining the individual discrimination to be based on identity only (Figure 1).

The two paradigms were presented here to the same 52 children, testing the hypothesis that the right hemispheric lateralization might be enhanced during individual face discrimination as compared to generic face categorization. In addition, we included a condition in the face individuation paradigm in which the same face stimuli were presented upside‐down. In adults, this manipulation greatly reduces the individual face discrimination response (Liu‐Shuang et al., 2014, 2016; Vettori et al., 2019), in line with the well‐known behavioral face inversion effect (Yin, 1969; for review see Rossion, 2008). Given that this behavioral effect of inversion is either absent in children (e.g., 6‐ and 8‐year‐old children: Carey & Diamond, 1977; Hills & Lewis, 2018; Schwarzer, 2000) or apparent but largely reduced as compared to adults (Carey, 1981; de Heering, Rossion, & Maurer, 2012), we expected no or a relatively small inversion effect of the electrophysiological index of individual face discrimination here, providing a platform to study the evolution of this effect during development.

## 2 MATERIALS AND METHODS

### 2.1 Participants

In total, 52 children (24 males, mean age = 5.56 years; range = 5.01–5.98 years), with normal/corrected‐to‐normal vision, were tested after the parents gave informed consent for a study approved by the Biomedical Ethical Committee of the University of Louvain. Two other children were excluded because of extremely noisy data on all electrodes or on posterior electrodes. Children were recruited from two schools of high‐socioeconomic status (Brabant‐Wallon region, Belgium). In total, 43 were Caucasian from different regions of Europe, five were from Middle‐Orient, three from mixed Caucasian‐African parents and one was African. They were unaware of the goal of the experiment and that a change of stimulus type occurred at a periodic rate during stimulation.

### 2.2 Behavioral testing

Children underwent a screening battery with subtests of the WISC‐R (cubes and codes), selective attention, verbal memory span, and reading competencies (grapheme‐phoneme production and recognition). They also participated in an independent experiment involving the presentation of letter strings, reported elsewhere (Lochy, Van Reybroeck, & Rossion, 2016). Details of testing and results are depicted in Table 1.

**Table 1:** Behavioral scores in the general cognitive functions’ assessment battery (*N* = 52)

Behavioral tests and subtests	Scores		
	Min	Max	Mean (*SD*)
General cognitive functions			
Visuo‐spatial reasoning (WISC‐IV, Block Design, standard note^a^)	1	16	8.94 (3.09)
Processing speed (WISC‐IV, Cancellation, standard note^a^)	1	19	9.5 (4.06)
Processing speed (WISC‐IV, Coding, standard note^a^)	1	16	7.58 (3.87)
Verbal span simple pseudowords (BELEC, CV syllables)	1	5	4.56 (0.85)
Verbal Span complex pseudowords (BELEC, CCV syllables)	1	4	2.52 (0.67)
Reading			
Production total score (/52)	0	42	7.19 (8.14)
Letters (/26)	0	22	6.31 (5.66)
Simple syllables (/11)	0	8	0.48 (1.63)
Complex syllables (/15)	0	12	0.40 (1.92)
Letter recognition (/26)	1	24	10.4 (6.25)

a Standard notes for the WISC subtest are calculated in reference to the youngest available norm in this test, that is, 6 years old children (1 year older than our sample). There are no norms for the reading tasks as children start reading instruction one year later.

### 2.3 EEG testing stimuli

#### 2.3.1 Individual face discrimination

Full‐front colored photographs of 25 male and 25 unfamiliar female faces from Caucasian young adults with a neutral expression (originally described in Laguesse, Dormal, Biervoye, Kuefner, & Rossion, 2012), taken under standardized conditions with respect to lighting, background, and distance from the camera were used (Liu‐Shuang et al., 2014; Figure 1a).

External features, such as hair and ears, were cropped out using Adobe Photoshop, and the isolated faces were put against a neutral gray background. Images were equalized for luminance (weighted RGB values) in Matlab (Mathworks). Final images were resized to a height of 250 pixels (width 186 ± 11 pixels). At a distance of 1 m, displayed with an 800 × 600 pixel resolution, they had an average size of 6.53 × 4 degrees of visual angle. Images were presented either in upright orientation (UP) or in flipped 180° for the inverted orientation (INV).

#### 2.3.2 Generic face categorization

In total, 250 images of various objects (animals, plants, man‐made objects) and 50 images of faces collected from the Internet were used (see Rossion et al., 2015). They differed in terms of color, viewpoint, lighting conditions, and background (Figure 1a). They were equalized in terms of luminance and contrast in Matlab (Mathworks). However, this normalization being applied to whole images, the individual faces and objects within the images remained highly variable in luminance and contrast (see Rossion et al., 2015). They were all resized to 200 × 200 pixels and shown in the center of the screen at an 800 × 600 pixel resolution. At a testing distance of 1 m, they subtended approximately 5.2 × 5.2 degree of visual angle.

### 2.4 Procedure

Each stimulation sequence started with a fixation cross displayed for 2–5 s, 2 s of gradual stimulation fade in, 40 s of stimulation sequence, and 2 s of gradual fade out. Stimuli were presented by means of sinusoidal contrast modulation at a base frequency rate of 6 Hz (i.e., one item every 166.66 ms, hence each item reached full contrast after 83 ms; Figure 1b). Given that the stimulus can be recognized at very low contrast (i.e., 20% or less), the actual duration of stimulus visibility is close to 140 ms. MATLAB 7.8 (The Mathworks) with PsychToolbox (Brainard, 1997) see http://psychtoolbox.org/) was used for stimulus display.

In each condition, every sequence had the same structure: base stimuli were presented at 6 Hz, and every fifth item was a stimulus of the contrasted category (frequency of 1.2 Hz, thus every 833 ms; Figure 1a).

In the generic face categorization paradigm, base stimuli were constituted of nonface objects (O) with faces (F) appearing every five items (OOOOFOOOOFOOOOF…). In the individual face discrimination paradigm, one randomly selected identity (A) among the 25 faces per gender constituted the ‘base’ stimulus. This base stimulus was repeated with random size variations at every cycle for that sequence, and the rare stimuli were other identities of the same gender (B, C, D, etc.) appearing every five items (AAAABAAAACAAAAD…). Each orientation condition (UP/INV) contained one sequence of female faces, and one sequence of male faces, for a total of 2 × 40 s stimulation per orientation. The sequence was repeated once for a total of 2 × 40 s.

A pause of about 30 s was done between each of the stimulation sequences, which were initiated manually to ensure low‐artifact EEG signals. The order of the two paradigms was counterbalanced across participants.

During stimulation, children fixated a central cross and were instructed to press the space bar for any brief (200 ms) color change of the fixation cross (blue to red; six changes randomly timed per sequence). The task's goal was to maintain a constant level of attention throughout the stimulation. Children performed this task almost at ceiling (91%–95%), showing high attention to the stimulation.

### 2.5 EEG acquisition and preprocessing

Children were seated comfortably at 1 m from the computer screen in a quiet room of the school. EEG was acquired at 1,024 Hz using a 32‐channel Biosemi Active II system (Biosemi), with electrodes including standard 10–20 system locations (http://www.biosemi.com). The magnitude of the offset of all electrodes, referenced to the common mode sense (CMS), was held below 50 mV. All EEG analyses were carried out using Letswave 5 (http://nocions.webnode.com/letswave), and Matlab 2012 (The Mathworks). After FFT band‐pass filtering around 0.1 and 100 Hz, EEG data were segmented to include 2 s before and after each sequence, resulting in 44‐s segments (−2 to 42 s). Data files were then resampled to 250 Hz to reduce file size and data processing time. Noisy channels were replaced using linear interpolation, with a total of 52 channels (no more than two electrodes for each participant, and never on posterior electrodes of interest). Finally, in six cases, we decided to keep only one epoch (instead of two) when the data was not possible to interpolate (for three children in the generic face categorization task, in one child in the upright face discrimination, and for two children in the inverted face discrimination task. All channels were re‐referenced to the common average. EEG recordings were then segmented again from stimulation onset until 39.996 s, corresponding exactly to 48 complete 1.2 Hz cycles within stimulation. This corresponds to the largest amount of complete cycles of 833 ms at the categorical change frequency (1.2 Hz) within the 40 s of stimulation period.

### 2.6 Frequency domain analysis

For each paradigm, the two trials of each condition were averaged in the time domain for each individual participant, in order to increase SNR. A Fast Fourier Transform (FFT) was applied to the averaged time‐window, and normalized amplitude spectra were extracted for all channels. This yielded EEG spectra with a high‐frequency resolution (1/39.996 s = 0.025 Hz), increasing SNR and allowing unambiguous identification of the response at the exact frequencies of interest (i.e., 6 Hz for the base stimulation rate and 1.2 Hz and its harmonics for the oddball stimulation). To quantify the responses of interest in microvolts for further analysis, the average voltage amplitude of the 20 surrounding bins, 10 on each side (i.e., the noise) was subtracted out, excluding the immediately adjacent bin (e.g., Retter & Rossion, 2016).

Based on previous responses in these paradigms in adults and visualization of the present data, we defined the harmonics to consider as of *Z* > 1.64 (*p* < .05, signal > noise) on one of the six contiguous lateral posterior channels (P7, P8, PO3, PO4, O1, and O2). We used a rather liberal statistical threshold given that it is better to include weak harmonic responses in the total amplitude response than fail to include genuine responses. For the generic face categorization paradigm, responses were significant on at least one of these channels up to the 14th harmonics (16.8 Hz). For the upright individual discrimination condition, responses were significant on at least one of these channels – in fact mainly P7 or P8 – up to the 9th harmonics (10.8 Hz). For the inverted individual discrimination condition, responses were significant on at least one of these channels – also mainly P7 or P8 – up to the 4th harmonic only (4.8 Hz). Finally, for the base rate response (6 Hz and harmonics), responses were significant up to the 7th harmonic (42 Hz).

In order to quantify the periodic discrimination response distributed on several harmonics, the baseline‐subtracted amplitudes of significant harmonics (excluding the base stimulation frequency) were summed for each participant, paradigm, and condition (Retter & Rossion, 2016). For a fair comparison, we compared upright and inverted face discrimination conditions by summing amplitude values across nine harmonics for both conditions (note that this does not provide any unfair advantage for the upright condition: responses across all harmonics should be considered for a complete quantification, and if a response is not above baseline for a given harmonic, that is, signal = noise, this corresponds to adding zeros). For the face categorization paradigm, we summed baseline‐subtracted amplitudes from the 1st to the 14th harmonic (excluding base rate).

The same was done to quantify the base rate response where we summed responses from the first (6 Hz) up to the 7th harmonic (42 Hz).

## 3 RESULTS

### 3.1 Visual discrimination responses

**Figure 2:**
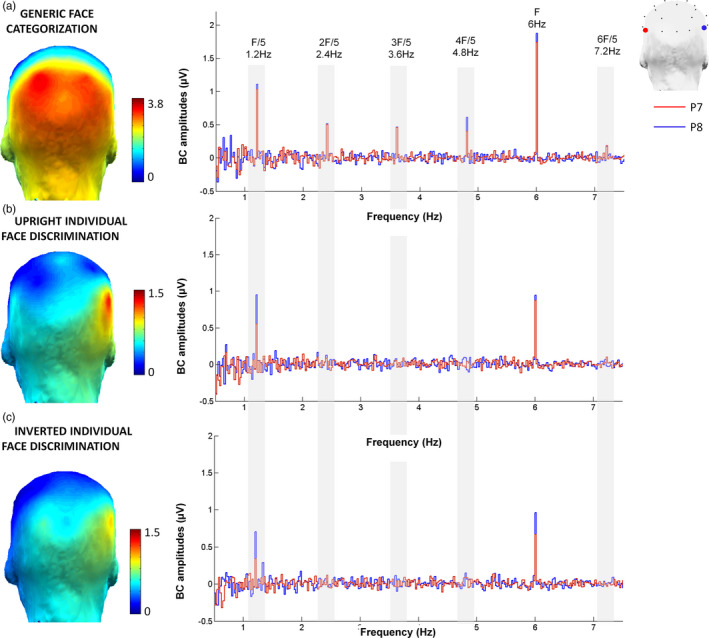
Discrimination responses to faces as a function of discrimination level (individual face discrimination or generic face categorization) in 5‐year‐old children (*N* = 52), for (a). Generic face categorization: faces versus objects, (b). Individual face discrimination for upright faces, and C. Individual face discrimination for inverted faces. Each row displays the left (in red) and right (in blue) lateral channels. The peaks on the spectra represent the response [baseline corrected (BC) amplitudes, see Methods] at the different frequencies of interest (base rate at 6 Hz (F), and discrimination of faces at 1.2 Hz (F/5) and harmonics (2F/5, 3F/5, etc.), as well as the scalp topographies of the discrimination responses

**Figure 3:**
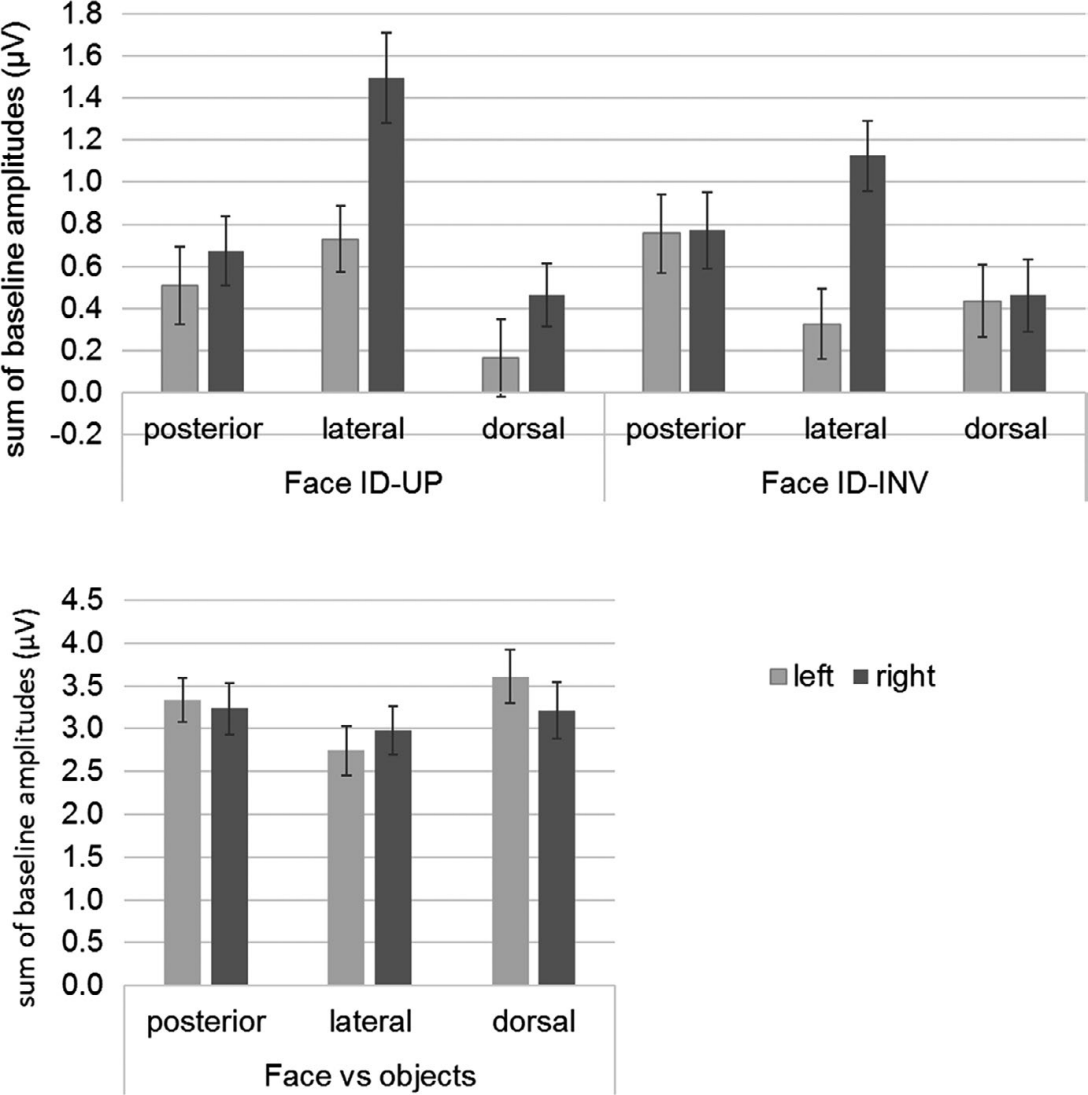
Histograms of amplitudes (µV) for discrimination responses in each paradigm, per hemisphere (left: light gray; right: dark gray) and electrode (posterior: O1/O2; lateral: P7/P8; dorsal: PO3/PO4). The top row plots responses in the individual face discrimination paradigm for upright (left panel) and inverted (right panel) faces. The bottom row displays the strong bilateral discrimination responses in the generic face categorization paradigm

**Figure 4:**
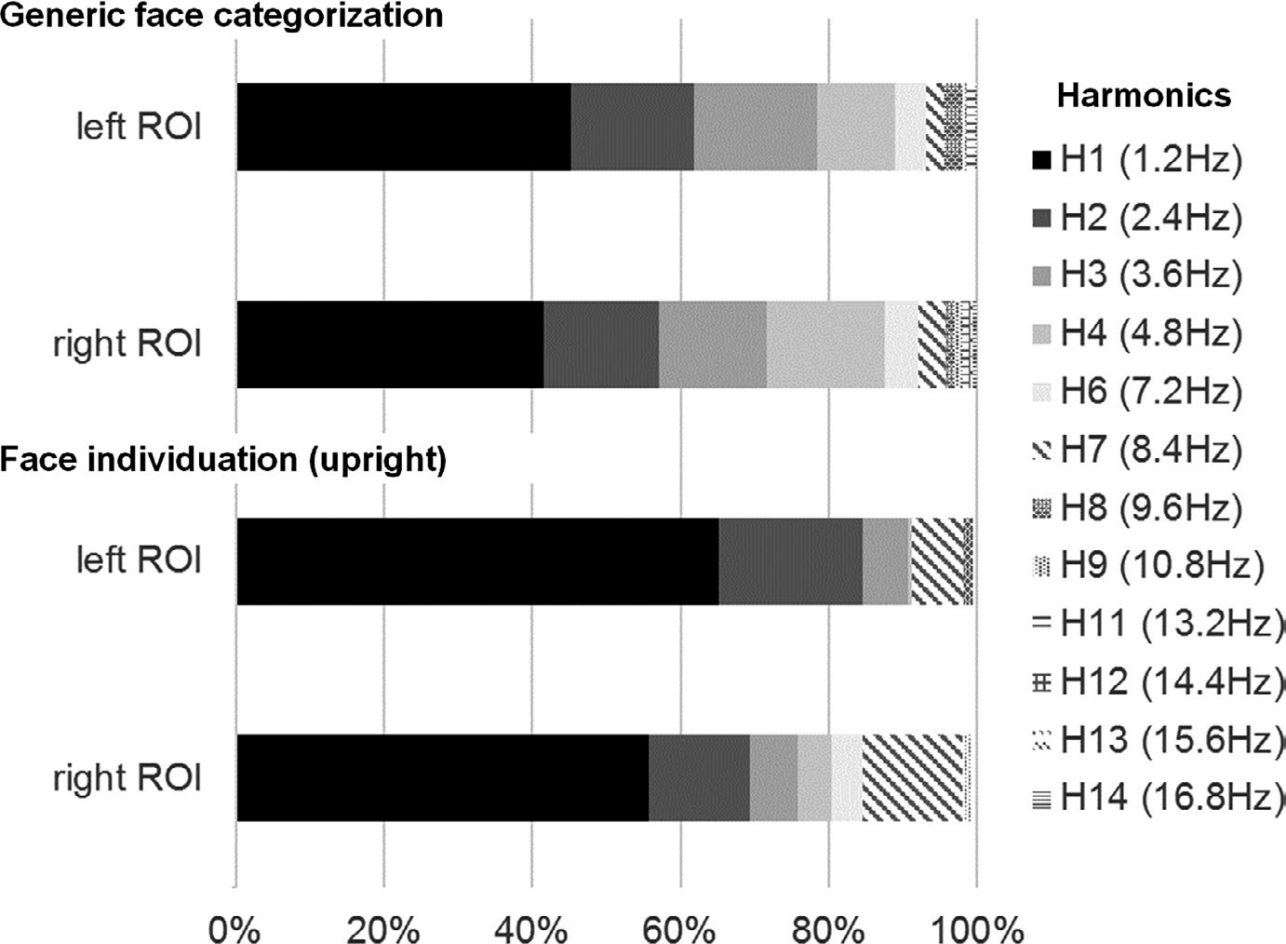
Distribution of discrimination responses over harmonics. In the generic face categorization paradigm (top part), the first harmonic represents 40%–45% of the total response, while in the individual face discrimination for upright faces (bottom part), the response is less distributed, the first harmonic representing about 60% of the total response

Scalp topographies and EEG spectra of grand‐averaged data showed a clear increase of signal (i.e., SNR > 1.7; sum of baseline‐corrected amplitudes >1.4 µV) in both paradigms (see Figures 2 and 3). However, there were two major differences between the two paradigms. First, in the individual face discrimination paradigm, the response was mainly located on electrode P8 (right lateral site), while in the generic face categorization paradigm, the response was bilateral and spread over dorsal (PO3, PO4), lateral (P7, P8), and posterior sites (O1, O2; Figure 2 for response spectra and topographies and Figure 3 for bar graph). Second, the neural response in the individuation paradigm concentrated on the first harmonic, which accounted for 60% of the total response. In contrast, the response was distributed over several harmonics in the generic face categorization paradigm, that is, the first harmonic accounting only for 40% of the response (see Figure 4).

For sake of comparison between the two paradigms, we included the three electrode sites in each hemisphere in the analysis.

#### 3.1.1 Generic face categorization

An ANOVA was computed on the sum of baseline corrected amplitudes at significant harmonics (from 1.2 Hz to 15.8 Hz, excluding the base rate at 6 Hz, 12 Hz), with the factors *Hemisphere* (left, right) and *Electrode Position* (lateral, posterior, dorsal) as repeated measures. There was no main effect of *Hemisphere* (*F* < 1; left: 3.229 µV, *SE*: 0.210; right: 3.142 µV; *SE*: 0.232), and no main effect of *Electrode Position* [*F*(2,102) = 2.202, *p* = .121] (lateral: mean = 2.863 µV; *SE* = 0.229; posterior: mean = 3.283 µV; *SE* = 0.215; dorsal: mean = 3.409 µV; *SE* = 0.277). The interaction between these two factors was not significant [*F*(2,102) = 1.251; *p* = .287].

#### 3.1.2 Individual face discrimination

An ANOVA was computed on the sum of baseline corrected amplitudes from 1.2 to 10.8 Hz, with *Orientation* (upright, inverted), *Hemisphere* (left, right), and *Electrode Position* (lateral, posterior, dorsal) as repeated measures.

We found a significant main effect of *Hemisphere* [*F*(1,52) = 11.496; *p* = .001], responses being overall larger in the right (mean: 0.832 µV, *SE*: 0.105) than in the left (mean: 0.487 µV, *SE*: 0.111) hemisphere. There was also a significant main effect of *Electrode Position* [*F*(1,52) = 14.214; *p* < .000], with larger responses on lateral (mean: 0.919 µV, *SE*: 0.103) than on posterior (mean: 0.677 µV, *SE*: 0.121) or dorsal (mean: 0.383 µV, *SE*: 0.111) electrodes. Finally, there was an interaction between *Electrode Position* and *Orientation* [*F*(2,102) = 4.652; *p* = .012], and between *Electrode Position* and *Hemisphere* [*F*(2,102) = 10.013; *p* < .000]. No other effects were significant (*F* < 1).

We decomposed these interactions by running two‐way ANOVAs by *Electrode Position*, with repeated measures on *Hemisphere* (left, right) and *Condition* (upright, inverted).

At lateral electrodes (P7, P8), there was a significant effect of *Hemisphere* [*F*(1,51) = 6.304; *p* = .015], responses being larger in the right (mean: 1.113 µV, *SE*: 0.138) than in the left hemisphere (mean: 0.528 µV, *SE*: 0.120). The main effect of *Orientation* was also significant [*F*(1,51) = 18.933; *p* < .000], responses being larger for upright (mean: 1.311 µV, *SE*: 0.153) than inverted faces (mean: 0.726 µV, *SE*: 0.119), with no interaction (*F* < 1; Figure 3).

At posterior electrodes (O1, O2), as well as at dorsal electrodes (PO3, PO4), there were no significant effects or interactions (Posterior: all *F*s < 1; dorsal: *Orientation* [*F* < 1], *Hemisphere* [*F*(1,51) = 1.611; *p* = .210], *Hemisphere* × *Condition* [*F* < 1]).

### 3.2 Hemispheric lateralization of the responses

In Table 2, we report the amplitude values of the responses in the left and right ROIs per paradigm and condition. On these values, we computed the percent increase of the response in the RH relative to the left hemisphere (LH) (as follows: 100*(RH‐LH)/LH) for each condition. We also calculated the lateralization scores (RH‐LH) and lateralization indexes (ranging between −1 and +1, as (RH‐LH)/(RH + LH)).

**Table 2:** Response amplitudes, lateralization scores and lateralization indexes in left and right ROIs in the different tasks and conditions

Task and condition	Left ROI (µV)	Right ROI (µV)	% increase in right	Lateralization score (R‐L)	Lateralization index (R‐L/R + L)
Generic face categorization	3.229	3.142	−2	−0.087	−0.01
Upright face individuation	0.469	0.878	87	0.409	0.30
Inverted face individuation	0.506	0.786	55	0.280	0.22

ROI, region‐of‐interest.

These indexes clearly show that children have a strong lateralized response only in the individual face discrimination paradigm for upright faces, with 87% increase of the response in the RH compared to the LH (LI of 0.3) and not at all in the generic face categorization paradigm. They also show response lateralization for inverted faces, although to a lesser extent than for upright faces (55% increase in the RH; but there was no significant interaction between *Orientation* and *Hemisphere* in the main analysis described above).

In the individual face discrimination conditions, we computed the strength of the FIE as the percent increase of the response for upright faces relative to inverted faces in each hemisphere, as follows: 100*(Up‐Inv)/Inv. Children responded only slightly more for upright than for inverted faces: in the RH, the FIE represented 11.7% increase of the average response for upright (0.878 µV) relative to inverted faces (0.786 µV), and in the LH, there was even a reverse trend – that is, a decrease – of 7% of the average response for upright (0.469 µV) relative to inverted faces (0.506 µV).

### 3.3 Correlational analyses

**Figure 5:**
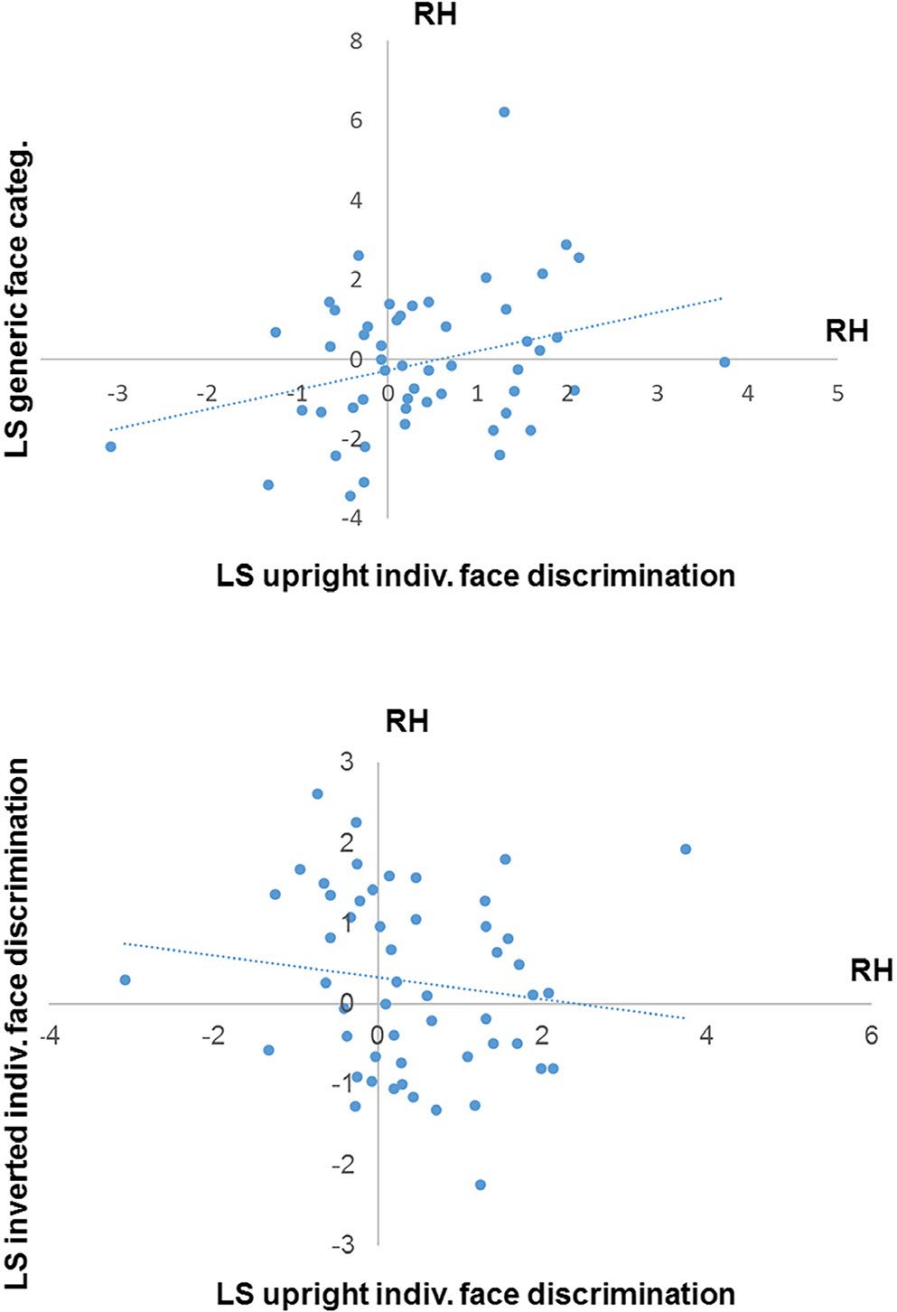
Relationship between lateralization scores for face processing. The correlation is significant between generic face categorization and upright individual face discrimination (top) and not significant between upright and inverted individual face discrimination (lower panel)

We found a positive correlation between lateralization scores for generic face categorization and individual face discrimination at upright orientation (Spearman *ρ* = 0.26, *p* = .03; see Figure 5a, top panel), and a near‐significant negative relationship between lateralization scores for upright and inverted faces in the individuation conditions (*ρ* = −0.221; *p* = .06).

No significant correlation emerged between letter naming or recognition scores and lateralization scores for generic face categorization (respectively, *ρ* = 0.10; *p* = .24 and *ρ* = 0.183; *p* = .09), lateralization scores for upright individual face discrimination (respectively, *ρ* = −0.08; *p* = .28 and *ρ* = −0.177; *p* = .11), or inverted individual face discrimination (respectively, *ρ* = 0.058; *p* = .34; *ρ* = 0.182; *p* = .09). No significant correlation with EEG measures was found either when considering the total production score (see Table 1; all *p* > .2).

### 3.4 Base rate responses

**Figure 6:**
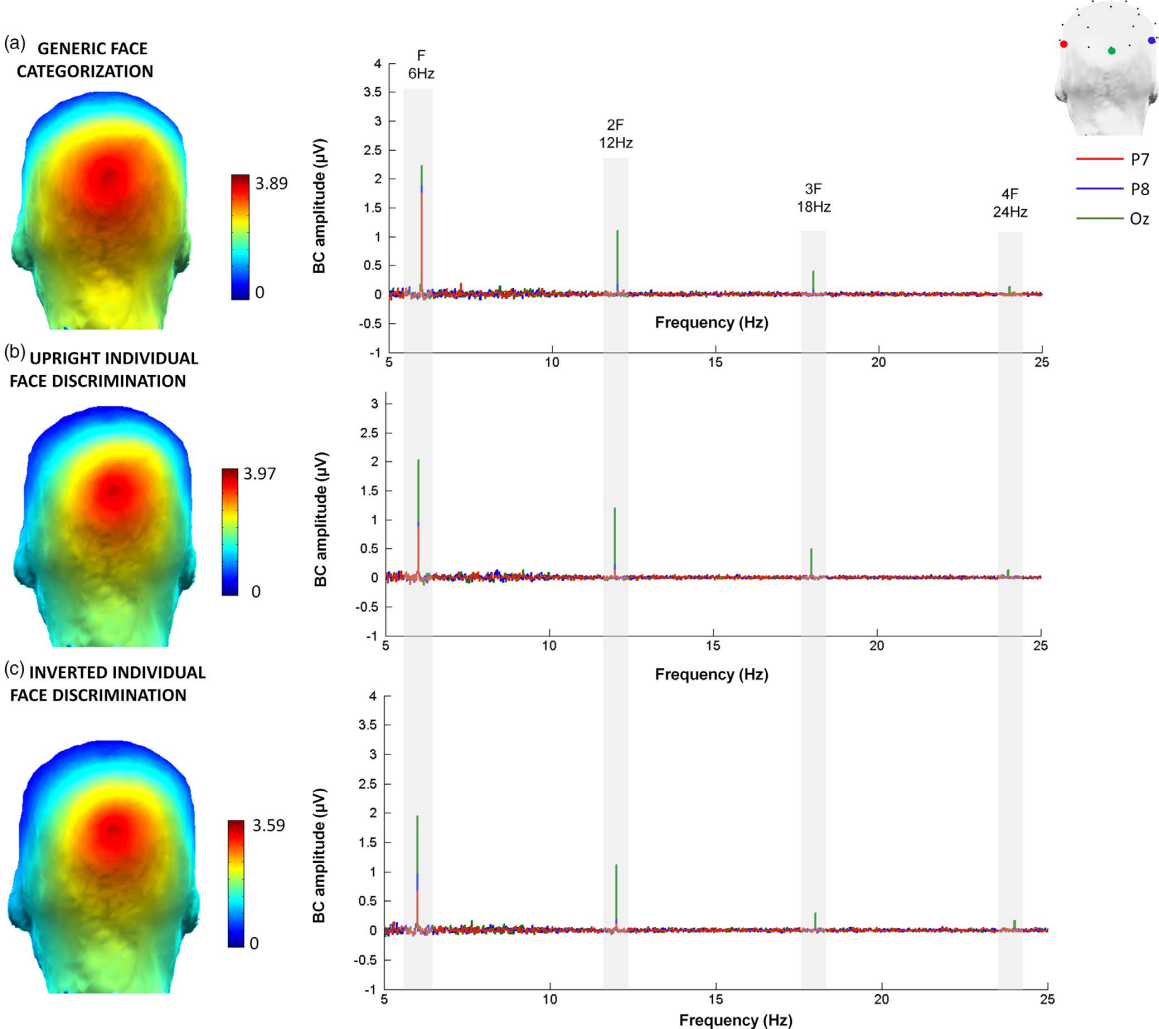
General base rate responses (visual synchronization to the base stimulation frequency) in each condition in 5‐year‐old children (*N* = 52), for (a). Generic face categorization: faces versus objects, (b). Individual face discrimination for upright faces, (c). Individual face discrimination for inverted faces. Each row displays the occipital‐middle (in green, Oz), the left (P7, in red), and right (P8, in blue) lateral channels. The peaks on the spectra represents the response [baseline corrected (BC) amplitudes, see Materials and Methods] at the main base rate frequency at 6 Hz (F), harmonics (2F, 3F, etc.), as well as the scalp topographies of the base rate responses

The scalp topography of base rate responses was very different from the face discrimination responses (Figure 6). In all three conditions, the strongest response was observed on middle occipital channel Oz, followed by O2 and O1. To perform analyses, we summed the baseline corrected amplitudes from base rate responses from 6 up to 42 Hz (seven harmonics). We then analyzed the summed corrected amplitudes separately for each paradigm, as for discrimination responses.

#### 3.4.1 Generic face categorization

An ANOVA was computed on the sum of baseline corrected amplitudes at significant harmonics (from 6 to 42 Hz), with the factors *Hemisphere* (left, right) and *Electrode Position* (lateral, posterior, dorsal) as repeated measures. There was a main effect of *Electrode Position* *F*(2,102) = 13.180, *p* < .000] (lateral: mean = 1.977 µV; *SE* = 0.160; posterior: mean = 3.132 µV; *SE* = 0.233; dorsal: mean = 2.689 µV; *SE* = 0.210), no main effect of *Hemisphere* *F*(2,102) = 2.259, *p* = .14] (left: 2.474 µV, *SE*: 0.176; right: 2.725 µV; *SE*: 0.177), and no interaction between these two factors [*F*(2,102) = 1.017; *p* = .36]. Paired *t* tests revealed that posterior electrodes (O1, O2) had a stronger response than lateral electrodes (P7, P8) (*t*(51) = 4.74, *p* < .000) and nearly stronger than dorsal electrodes (PO3, PO4) (*t*(51) = 1.894; *p* = .06). Dorsal electrodes also displayed a stronger response than lateral electrodes (*t*(51) = −3.543; *p* = .001).

#### 3.4.2 Individual face discrimination

An ANOVA was computed on the sum of baseline corrected amplitudes from 6 to 42 Hz, with *Orientation* (upright, inverted), *Hemisphere* (left, right), and *Electrode Position* (lateral, posterior, dorsal) as repeated measures.

We found a significant main effect of *Hemisphere* [*F*(1,51) = 8.316; *p* = .006], responses being overall larger in the right (mean: 2.140 µV, *SE*: 0.16) than in the left (mean: 1.766 µV, *SE*: 0.125) hemisphere. There was also a significant main effect of *Electrode Position* [*F*(1,51) = 56.825; *p* < .000], with larger responses on posterior (mean: 2.751 µV, *SE*: 0.198) than on dorsal (mean: 2.057 µV, *SE*: 0.166) or lateral (mean: 1.052 µV, *SE*: 0.091) electrodes (all *t* tests significant at *p* = .000). Finally there was also a main effect of *Orientation* [*F*(1,51) = 7.989; *p* = .007], responses being stronger for upright faces (2.074 µV, *SE*: 0.177) than for inverted faces (mean: 1.832 µV; *SE*: 0.123). Contrary to discrimination responses, there were no interactions between these factors.

For sake of comparison with the individual discrimination response, which differed only between upright and inverted faces on lateral electrodes P7 and P8, we contrasted the base rate response also on these electrodes. There was no effect of *Orientation* [*F*(1,51) = 1.369; *p* = .247], a non‐significant trend for *Hemisphere* [*F*(1,51) = 3.835; *p* = .06], and no interaction between these factors [*F*(1,51) = 2.005; *p* = .16].

## 4 DISCUSSION

Coupling FPVS with EEG, we found a robust neural index of individual face discrimination in preschool children, this response being much (i.e., almost twice) larger in the right as compared to the left hemisphere. To our knowledge, this is the first electrophysiological evidence of a right hemispheric advantage in face perception in preschool children: previous studies relying on standard ERP measures, mainly of the face‐sensitive N170 component, did not report significant right lateralization in children of various age‐groups until late adolescence (Dundas et al., 2012, 2014; Kuefner, 2010; Taylor, Mills, Zhang, & Pang, 2010). However, these studies did not measure individual face discrimination, rather considering the raw EEG response to face images, or the difference between faces and nonface stimuli. Here, as in our previous report with a subset of the children tested in the present study (Lochy et al., 2019), we confirm with FPVS‐EEG that the discrimination between faces and nonface stimuli (objects of various categories) does not elicit any right hemispheric lateralization in the very same preschool children showing this lateralization effect during individual face discrimination. The difference observed here in the right hemispheric lateralization obtained in the same population of children with two different paradigms measuring neural responses to faces indicates that the level of (visual) discrimination is a key factor to characterize and understand hemispheric lateralization of face processing.

As presented in the introduction, the right hemispheric dominance for individual face discrimination but not for generic face categorization in preschool children is in line with a variety of observations made in adults, showing either enhanced or exclusive right lateralization when individuating faces as compared to the categorization of faces as faces. This is the case for EEG measures, either obtained in standard ERP paradigms (e.g., Jacques & Rossion, 2007) or during FPVS (Liu‐Shuang et al., 2016; Rossion et al., 2015) and, most importantly, when considering the interruption of function either following brain damage (e.g., Rossion et al., 2011), transcranial magnetic stimulation over the right lateral occipital cortex (Ambrus et al., 2017; Pitcher et al., 2007; Solomon‐Harris et al., 2013) or intracranial stimulation in the right inferior occipital gyrus and fusiform gyrus (Chong et al., 2013; Jonas et al., 2012, 2015; Keller et al., 2017).

The right hemispheric dominance for individual face discrimination in preschool children contradicts the view that the right hemisphere becomes dominant for face processing only during late childhood or adolescence due to competition in the left hemisphere with visual representations of letters and words following reading acquisition (Behrmann & Plaut, 2013; Dehaene et al., 2010; Dehaene‐Lambertz, Monzalvo, & Dehaene, 2018). Although our findings do not exclude a role of reading acquisition in increasing right lateralization for face processing in general, the lack of significant correlation between letter naming or recognition scores and right hemispheric lateralization in either of the paradigms in our large sample also fails to support this view. Rather, our observations are in line with the view that the right hemispheric specialization appears early in life (Adibpour et al., 2018; de Heering & Rossion, 2015; de Schonen et al., 1986; de Schonen & Mathivet, 1989, 1990; Otsuka, 2014), independently of reading acquisition. However, notably, if early individual face discrimination in infants also appears to be strictly right lateralized (Adibpour et al., 2018), strong right hemispheric lateralization effects in infants are observed even for face versus object discrimination (de Heering & Rossion, 2015; Otsuka, 2014). Altogether, these observations indicate, as we suggested previously, that other factors than reading acquisition, such as the late maturation of the corpus callosum (de Schonen & Bry, 1987; Le Grand et al., 2003; Liegeois, Bentejac, & De Schonen, 2000) modulate lateralization of face processing between infancy and early childhood (Lochy et al., 2019). The reason why, contrary to generic face categorization, individual face discrimination does not bilateralize between infancy and early childhood may be because this challenging function is still very limited in infancy, and undergoes a long developmental course. This view is supported by several observations regarding the nature of the individual face discrimination in preschool children in the present study.

### 4.1 Rapid individual face discrimination in preschool children

The observation of a robust individual face discrimination response in young children is not trivial and allows broadening our knowledge about children's ability to individuate faces. Behavioral and electrophysiological studies suggest that even infants of a few days or months of age can discriminate images of individual faces (de Haan & Nelson, 1999; Pascalis & de Schonen, 1994; Peykarjou, Pauen, & Hoehl, 2015; Scott, Shannon, & Nelson, 2006; Turati, Bulf, & Simion, 2008), perhaps involving specifically the RH (Adibpour et al., 2018; de Schonen & Mathivet, 1990; Scott et al., 2006). However, it is fair to say that the evidence provided in these studies based on very few pairwise image discriminations (except in Peykarjou et al., 2015), and could be accounted for by low‐level visual cues, even when different head orientations are presented (Scott et al., 2006; Turati et al., 2008). Moreover, electrophysiological studies have sometimes failed to report any effect of individuation of faces in infants (Peykarjou, Pauen, & Hoehl, 2014; Peykarjou et al., 2015 for female faces) and when these effects are found they are generally inconsistent across studies.

Behavioral studies in young children (3–5 years old) report relatively weak performance even in simple two alternative forced‐choice individual face discrimination tasks (e.g., Hills & Lewis, 2018; Sangrigoli & De Schonen, 2004). But the extent to which this low performance level of young children is due to difficulties in individuating faces per se or to difficulties in task understanding, attention, or decision making processes remains unknown.

Although our paradigm also relies on full‐front repeated unfamiliar face images, it involves a large number of highly variable individual discriminations, that is, 25 faces are used in a given stimulation sequence, with each face stimulus appearing at 1.2 Hz differing in terms of specific features from the base face identity presented. Face stimuli are devoid of external features and all have a neutral expression and, importantly, they change substantially in size at every stimulation cycle to force individual face discrimination beyond simple image‐based cues (Dzhelyvova & Rossion, 2014). Moreover, each new face identity appears only for the time of one fixation (i.e., less than 200 ms) and is forward‐ and backward‐masked by the repeated face stimulus in the sequence, requiring rapid and challenging individual face discrimination. These characteristics, together with the observation of the individual face discrimination response over high‐level visual regions of the occipitotemporal cortex rather than over the medial occipital cortex (Liu‐Shuang et al., 2014; Figure 2 here) and the specific absence of a significant response in prosopagnosia following brain‐damage (Liu‐Shuang et al., 2016) indicate that the paradigm measures a high‐level visual discrimination response. Hence, the presence of a clear significant EEG response in the population of preschool children tested here supports the ability of the young human brain to individuate faces already at a certain level of expertise.

Nevertheless, several key aspects of the observed individual face discrimination response warrant further discussion. First, the relatively lower magnitude of the individual face discrimination response as compared to the discrimination of faces versus objects is understandable when considering how fine‐grained this discrimination is (i.e., physical differences between individual face images are small compared to physical differences between faces and objects). Interestingly however, the ratio between the amplitude obtained in the two paradigms (i.e., about 1/4th or 25% of signal in children: 3.142 µV and 0.878 µV) is not very different than the ratio observed in adults (Liu‐Shuang et al., 2016, about 30% of the signal in adults: 2.324 µV and 0.885 µV).

Second, while the face versus object discrimination response spreads over multiple harmonics of 1.2 Hz, the individual face discrimination response is mainly accounted for (about 60%) by the first harmonic in the EEG spectrum (Figures 2 and 4). This is clearly different than the adult response, which is distributed over several harmonics (Liu‐Shuang et al., 2014, 2016), with the first harmonic accounting for less than 20% of the response (from Liu‐Shuang et al., 2014). That is, these data indicate that the response is much more complex in adults than children, with a larger number of higher frequency components involved, providing a potentially useful qualitative marker of the human development of individual face discrimination. Since these harmonics reflect the nonlinearity of the individual face discrimination response (see Norcia, Appelbaum, Ales, Cottereau, & Rossion, 2015; Retter & Rossion, 2016), the same relative distribution of amplitude at the different harmonics, with a larger response in adults than children, would indicate merely a quantitative increase over development. However, a change in the distribution of the response among harmonics as found here points to a qualitative change with development rather than a mere increase due to general factors such as global processing efficiency or attention.

Third, while the generic face categorization response is widely distributed over all posterior electrode sites, the individual face discrimination response is much more focal and ventral (Figure 2). Although the neural circuits subtending these responses remain undetermined, the topographical difference between the two face discrimination levels seems to reflect the involvement of a broader versus a more specific neural system. In humans, face recognition is subtended by an extended cortical network of face‐selective regions, divided into a ventral and a relatively more dorsal component (Calder & Young, 2005; Duchaine & Yovel, 2015; Haxby, Hoffman, & Gobbini, 2000). Individual face discrimination is a key aspect of facial identity recognition, which depends essentially on ventral regions, that is, the inferior occipital gyrus, fusiform gyrus, and inferotemporal cortex (Duchaine & Yovel, 2015; Haxby et al., 2000; Rossion, 2014). It is not thought to rely on face‐selective regions of the superior temporal sulcus (STS), which are rather involved in coding changeable aspects of faces such as facial expression, eye gaze direction, or head orientation (Duchaine & Yovel, 2015; Haxby et al., 2000; Puce, Allison, Bentin, Gore, & McCarthy, 1998). Providing that these latter functions reach maturity earlier in development than face identity recognition, the children's cortical face‐selective network as a whole might be less driven by activity in the ventral system than in adults, accounting for the relatively broad and dorsal activity recorded in the generic face categorization task.

The fourth and last point to discuss concerns the effect of face inversion. Responses at the base rate (6 Hz and harmonics), which reflect a general synchronization to the visual stimulation (a mixture of low‐ and high‐level processes), reveal a decreased response to inverted faces. However, this general inversion effect does not reflect *individuation* of faces and is not significant on the lateral occipitotemporal electrodes capturing the largest individual discrimination responses. Regarding this response, there was a significant but relatively small reduction of amplitude to inverted faces (i.e., the face inversion effect) in preschool children.

The origin and developmental course of the face inversion effect – arguably the most reliable effect in human face recognition research ‐ remain controversial (Hills & Lewis, 2018; McKone, Crookes, Jeffery, & Dilks, 2012). On one hand, sensitivity to face inversion in measures of individual face discrimination is present throughout development (McKone et al., 2012). Indeed, a number of studies have shown that infants discriminate faces upright but fail to discriminate the same stimuli inverted (newborns, Turati et al., 2008; across view change in 4 months old, Turati, Sangrigoli, Ruel, & de Schonen, 2004). In young children, discrimination is better upright than inverted in both short‐ and long‐term memory tasks (see Hills & Lewis, 2018 for review). Based on these observations, two extensive reviews by McKone and colleagues have argued that the effect of face inversion – as an index of holistic face processing – is mature early, that is, by 5–7 years of age at the latest and possibly earlier (Crookes & McKone, 2009; McKone et al., 2012). However, against this claim, a number of behavioral studies have failed to find significant face inversion effects in young children (e.g., 6–8 years old: Carey & Diamond, 1977; Hills & Lewis, 2018; Schwarzer, 2000) or found a reduced effect as compared to adults (Carey, 1981; de Heering et al., 2012; Sangrigoli & De Schonen, 2004) with the effect increasing over childhood and adolescence (Carey & Diamond, 1977; de Heering et al., 2012; Hills & Lewis, 2018; Itier & Taylor, 2004).

Given that behavioral measures reflect a wide range of cognitive processes that undergo a long developmental course, and that different tasks and paradigms are used to test different populations, this controversy is difficult to resolve with behavioral studies alone. Here, with a quantitative electrophysiological measure that does not require an explicit face‐related task, we found not only that the individual face discrimination response is qualitatively different (i.e., simpler) than in adults, but that the inversion effect is much smaller: the EEG index of individual face discrimination was only 11% larger in amplitude for upright than inverted faces in the preschool children tested here, while it is about two times larger for upright than inverted faces in 8‐ to 12‐year‐ old children (Vettori et al., 2019) and almost two and half times larger in adults (Liu‐Shuang et al., 2014). This relative difference cannot be accounted for by an overall reduction of EEG amplitude, which is typically quite large in young children (e.g., Kuefner et al., 2010), including in the present study (i.e., the absolute amplitude of the individual discrimination response to upright faces (0.878 µV) was of 99% of the adult response (0.885 µV). Our observations therefore indicate that the inversion effect, although present in preschool children, is quantitatively smaller than in older children and adults, pointing to a large influence of experience with upright faces during social development that tunes the visual system to holistic processing specifically for this orientation. Given that the implicit measure used here is applicable to a wide age range without any change of paradigm, it should prove to be particularly useful in future studies to track the developmental course of individual face discrimination and its hemispheric lateralization, the face inversion effect, and face processing in general.

## CONFLICT OF INTEREST

None.

## Data Availability

The data that support the findings of this study are openly available from the corresponding author (Aliette Lochy, aliette.lochy@uni.lu) upon reasonable request.
