# Supplementary figures and images for: The right hemispheric dominance for face perception in preschool children depends on the visual discrimination level
Source: Dev Sci. 2019 Nov 15;23(3):e12914. doi: 10.1111/desc.12914 (PMC7379294; doi:10.1111/desc.12914)

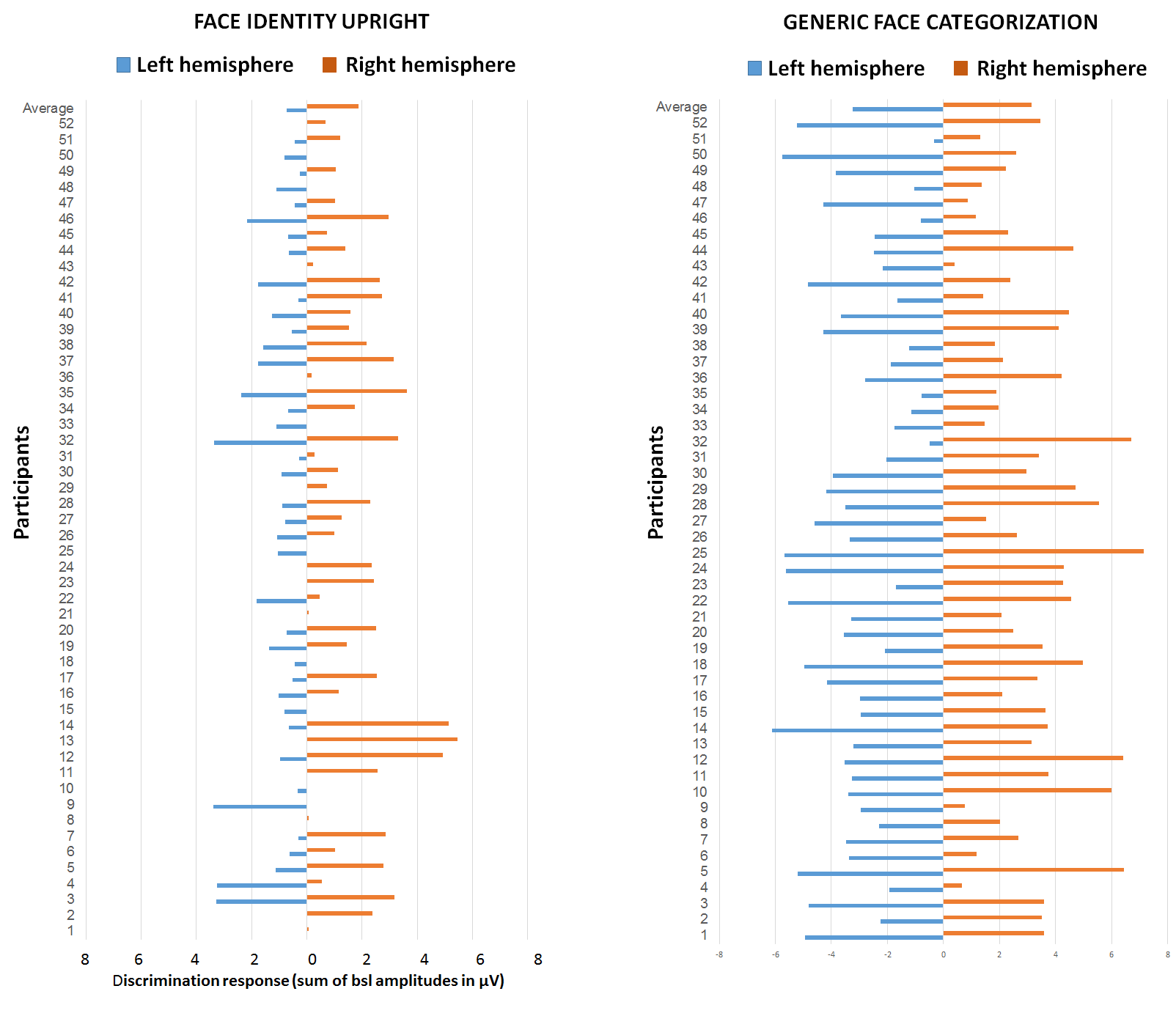

Supplement: Supplementary file 1 [file DESC-23-e12914-s001.png]
